# Hanging on the telephone: Maintaining visuospatial bootstrapping over time in working memory

**DOI:** 10.3758/s13421-023-01431-5

**Published:** 2023-06-06

**Authors:** Richard J. Allen, Jelena Havelka, Candice C. Morey, Stephen Darling

**Affiliations:** 1 School of Psychology, University of Leeds, Leeds, UK; 2 School of Psychology, Cardiff University, Cardiff, UK; 3 Division of Psychology, Sociology and Education, Queen Margaret University, Edinburgh, UK

**Keywords:** Working memory, Short term memory, Recall, Visuospatial bootstrapping, Maintenance

## Abstract

Visuospatial bootstrapping (VSB) refers to the phenomenon in which performance on a verbal working memory task can be enhanced by presenting the verbal material within a familiar visuospatial configuration. This effect is part of a broader literature concerning how working memory is influenced by use of multimodal codes and contributions from long-term memory. The present study aimed to establish whether the VSB effect extends over a brief (5-s) delay period, and to explore the possible mechanisms operating during retention. The VSB effect, as indicated by a verbal recall advantage for digit sequences presented within a familiar visuospatial configuration (modelled on the T-9 keypad) relative to a single-location display, was observed across four experiments. The presence and size of this effect changed with the type of concurrent task activity applied during the delay. Articulatory suppression (Experiment 1) increased the visuospatial display advantage, while spatial tapping (Experiment 2) and a visuospatial judgment task (Experiment 3) both removed it. Finally, manipulation of the attentional demands placed by a verbal task also reduced (but did not abolish) this effect (Experiment 4). This pattern of findings demonstrates how provision of familiar visuospatial information at encoding can continue to support verbal working memory over time, with varying demands on modality-specific and general processing resources.

## Introduction

Working memory is typically defined as a limited capacity system or ensemble of components that supports temporary storage and processing of information in the service of complex cognition and task goals (e.g., Baddeley et al., 2021; Cowan et al., 2021). Performance on any given working memory task is likely to reflect contributions from a variety of cognitive components, depending on the task, materials, and individual (Logie et al., 2021; Macken et al., 2015). Among a range of contributory components, this might include verbal, visual, spatial, and motor processing (Cowan et al., 2021; Li et al., 2022; Logie et al., 2021), along with support from pre-existing knowledge structures in long-term memory (LTM). Understanding how such representational and processing dimensions combine is important both in developing theoretical understanding and in identifying how working memory might be supported and enhanced.

**Fig. 1:**
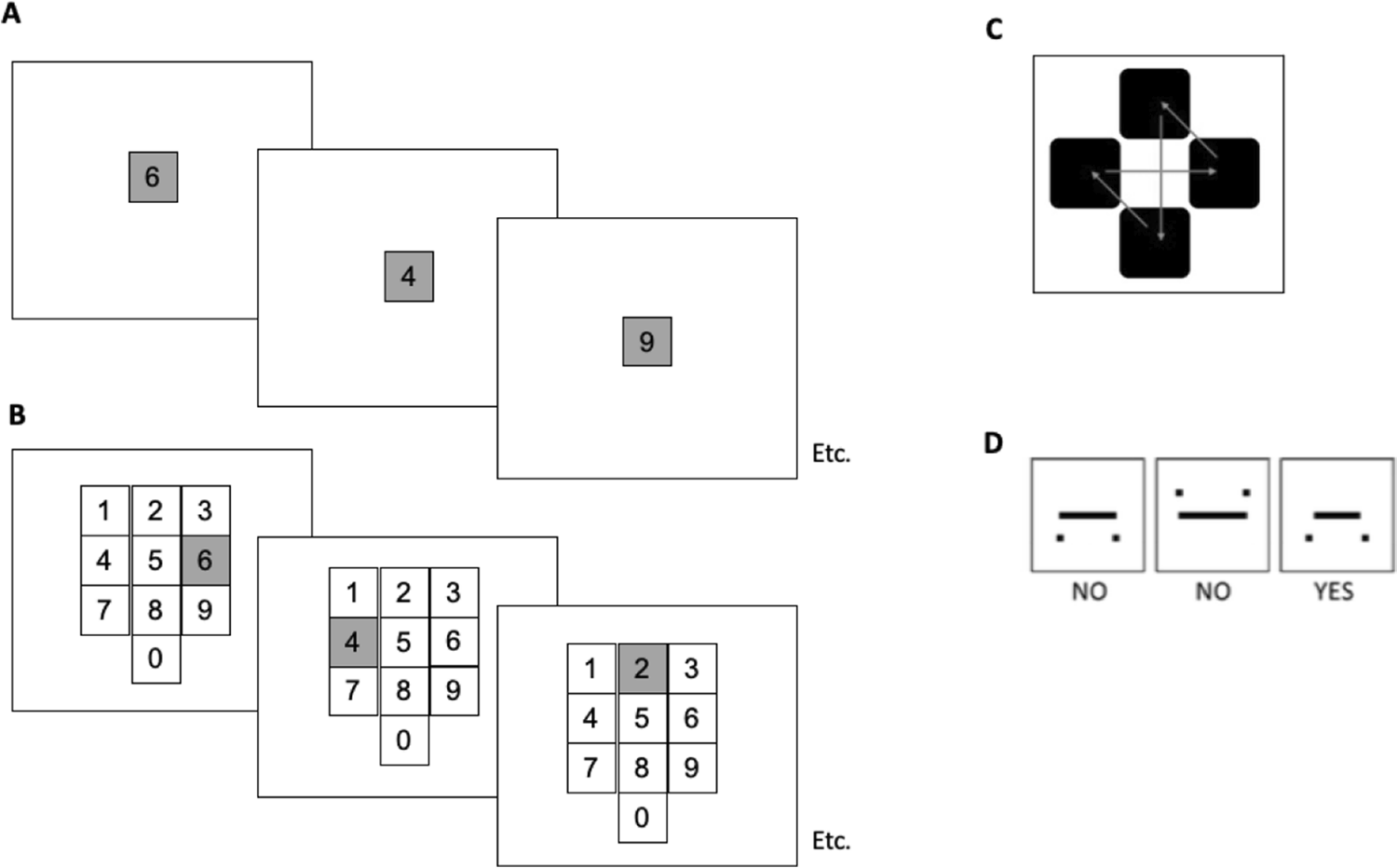
**A** Single location presentation condition. **B** Keypad presentation condition. **C** Spatial tapping pattern used in Experiment 2. **D** Examples of visuospatial stimuli used in Experiment 3. Images are show in grayscale and are not to scale

One phenomenon that may involve such a combination of components has been termed ‘visuospatial bootstrapping’ (VSB; for review, see Darling et al., 2017). This typically involves the demonstration that recall of digit sequences is improved when the digits are visually presented within a familiar spatialised display, namely a typical ‘keypad’ as often encountered on telephone displays and computer keyboards (see Fig. 1). A key feature of the effect is that any benefits of the visuospatial configuration is incidental to the task, with participants asked to verbally recall the visually presented digits in all conditions. The VSB effect was first established by Darling and Havelka (2010), who found that keypad presentation improved digit recall compared to use of a single location, or a horizontal number line. Darling et al. (2012) subsequently suggested that the presence of pre-existing representations concerning the verbal-spatial configuration was critical to the effect, finding that recall was not enhanced in the same way when digits were presented as randomised reconfigurations within the same overall display.

Following these early demonstrations of VSB, it has since been shown to be a robust and replicable effect that can be observed across different configurations and populations. The basic effect has also been extended from the typical keypad to a clockface configuration (Mallik et al., 2022). Children aged 9 years appear to show the keypad advantage in digit recall, though this effect was not observed in a younger group of children (aged 6 years), possibly reflecting developmental changes in working memory function and LT knowledge (Darling et al., 2014). The effect has also been shown in healthy older adults (Calia et al., 2015), indicating that aspects of cognitive function affected by age-related decline are not critical to the derivation of the VSB effect. Within a neuropsychological context, Race et al. (2015) found that amnesic patients with medial temporal lobe damage were able to exhibit a VSB effect, as well as a sentence superiority effect in recall (e.g., Baddeley et al., 2009), to the same extent as healthy controls.

The VSB phenomenon and the principles underlying it also appear to go beyond immediate memory and benefit longer term retention too. Darling et al. (2020) asked participants to recall a sequence of 15 digits that was repeatedly presented, with the aim of exploring learning of this supraspan sequence over time. Presentation within a typical keypad configuration led to faster sequence learning, compared to a single location condition. This was extended in a second experiment, with participants showing enhanced learning of nine-item nonword sequences when they were presented in a static visuospatial configuration, relative to a random changing pattern. Thus, embedding verbal material within a meaningful and/or consistent visuospatial context offers ways of enhancing both working memory and longer-term learning.

A core assumption underlying the VSB effect is the use of available visuospatial information to support or ‘bootstrap’ verbal memory performance. It connects to other illustrations of ‘spatialization’ phenomena in working memory, such as SNARC (Spatial-Numerical Association of Response Codes; Dehaene et al., 1993), and more recently SPoARC (Spatial-Positional Association of Response Codes; van Dijck & Fias, 2011). In the latter case, sequences of to-be-remembered items appear to have an internally driven left-to-right spatial dimension (see also Abrahamse et al., 2014, 2017; Guida et al., 2016, 2020). One difference is that these spatialization effects can be internally derived rather than environmentally dependent, whereas VSB typically hinges on explicit provision of visuospatial support in the environment, although Guida and Maherault (2021) have shown that this latter effect can be produced after training without an explicit configuration being presented to participants. Availability of pre-existing verbal-spatial mapping seems to be important, as evidenced by the advantage for typical over random displays (Darling et al., 2012, 2014). However, recall accuracy for novel, random configurations that are static across trials can improve over trials with sufficient repetition (Darling et al., 2012), and sequence learning can be enhanced by previous familiar spatial configurations (Darling et al., 2020). Similarly, Yousif et al. (2021) found that previously unfamiliar spatial structure can improve STM for visual objects (coloured shapes) when the spatial structure of displays is repeated during the session.

One approach to explore possible cognitive mechanisms underlying VSB is to adopt dual-task procedures. Allen et al. (2015) found that verbal and spatial tasks performed during encoding had opposite impacts on the VSB advantage. Articulatory suppression (AS) achieved by participants repeatedly producing a verbal phrase (*“coca-cola”*) concurrent with sequence presentation served to increase the advantage for typical keypad over single location digit recall, relative to a no-task condition. In contrast, a spatial tapping (ST) task in which participants use their finger to repeatedly tap out a simple spatial pattern removed the keypad advantage. This was only the case during encoding though; shifting ST to the recall phase did not impinge on the VSB effect. These findings suggest that initial encoding and storage of a to-be-remembered verbal sequence that incorporates meaningful spatial information draws on spatial processing, while reducing reliance on verbal coding. This dual-task approach was subsequently extended by Calia et al. (2019), who showed that manipulation of the executive-attentional demands placed by a concurrent verbal task did not reduce the advantage for typical keypad over single location recall, and in fact appeared to somewhat increase the effect. This finding is in keeping with the apparent incidental benefits of VSB and other forms of spatialisation to task performance (see also Yousif et al., 2021), and with broader observations of automatic feature binding in working memory (e.g., Allen et al., 2006, 2009; Baddeley et al., 2011).

The observation from Allen et al. (2015) that articulatory suppression serves to increase the VSB effect was subsequently replicated across experiments by Allan et al. (2018). However, this latter study also found that manipulating path complexity of the spatial pattern during digit presentation had equivalent impacts on typical and random keypad arrays. Allan et al. suggested that visuospatial information can be incorporated into a working memory representation even when it is random, and does not map onto pre-existing knowledge. However, it is of limited effectiveness in supporting the advantageous use of multi-modal processing without prior knowledge of the mappings between spatial and verbal content.

Studies to date have focused on processes engaged during the encoding and immediate retrieval of to-be-remembered sequences. While this necessarily involves storage of early items during presentation of later parts of the sequence, it is not possible to differentiate between encoding and maintenance phases of the task. As such, we cannot draw conclusions regarding the processes operating after encoding has been completed, when items are being retained in working memory for subsequent recall. Encoding and maintenance are distinct phases of any working memory task; encoding requires the creation, consolidation and serial updating of a novel, dynamically changing representation, while subsequent maintenance might reflect passive storage supplemented by active processes and strategies intended to support successful maintenance over time.

It is likely that different representational formats and maintenance methods are engaged depending on the type of material that is being retained in working memory. Verbal material is often assumed to be held as a domain-specific phonological representation (e.g., Baddeley et al., 2021; Jarrold et al., 2011), with visuospatial retention based on domain-specific (Baddeley et al., 2021; Logie et al., 2021) and/or domain-general (e.g., Morey, 2018; Morey & Miron, 2016) storage. Theoretical frameworks in working memory often also incorporate domain-general storage for the most recent or goal-relevant items, variously referred to as a focus of attention, focus of awareness, or episodic buffer (Baddeley et al., 2021; Barrouillet & Camos 2021; Cowan et al., 2021; Logie et al., 2021; Oberauer, 2021). Representations may then be kept active and forgetting reduced through the application of verbal rehearsal (e.g., Barrouillet & Camos 2021; Jarrold et al., 2011) and/or attentional refreshing (Atkinson et al., 2022; Barrouillet & Camos 2021; Sandry et al., 2020; Souza et al., 2018). There is also evidence that the requirement to make eye movements during retention has a detrimental impact on memory specifically for location-based information (e.g., Pearson & Sahraie, 2003; Postle et al., 2006), indicating disruption of spatial rehearsal or control processes during working memory maintenance. It is not clear at present, however, what kinds of representation and process might be recruited during the maintenance of verbal information that varies in spatial context during initial encoding. Understanding how VSB effects survive over time, and what processes and strategies might support memory for verbal sequences that are presented with or without useful visuospatial context, will aid understanding of working memory retention, and of how the kind of multimodal benefits conferred by VSB can best be supported in a practical context.

The present study involved a series of experiments that compared digit recall following presentation either in a single spatial location or in a familiar keypad configuration. Each experiment applied a different concurrent task during a post-encoding, pre-test delay, with the assumption that each task disrupts a particular set of cognitive processes. Changes in performance overall, but more importantly in the size of any advantage for keypad over single location presentation, would then reflect contributions from the processes being targeted in each case. Previous explorations of VSB in the working memory domain have implemented a brief (1-s) retention interval between sequence completion and the test phase. The present study extended this to 5 s to allow performance of concurrent tasks during maintenance. The first experiment extended Allen et al. (2015, Experiment 1; also, Allan et al., 2018) by applying articulatory suppression during the maintenance period to disrupt verbal processing. Experiments 2 and 3 then used spatial tapping and visuospatial line judgments respectively. Finally, Experiment 4 manipulated complexity of the verbal task during delay to target general executive control resources.

## Experiment 1

Experiment 1 began by examining contributions from verbal processing, using an articulatory suppression (AS) task added during the retention interval. This form of task has been commonly used in the working memory literature to block or at least substantially disrupt verbal recoding and rehearsal processes for visually presented materials (e.g., Allen et al., 2006; Baddeley et al., 1975, 2009; Mate et al., 2012; Morey & Cowan, 2005). In the context of VSB, Allen et al. (2015) had participants perform a simple AS task during encoding and found that while it negatively affected both display conditions, this was substantially larger for single location than keypad trials, a finding that was subsequently replicated across separate experiments by Allan et al. (2018). This was interpreted as reflecting a reduced reliance on verbal processing when useful spatial information is also available.

Previous work has shown that maintenance of verbal material over time draws on domain-specific verbal processing (e.g., Jarrold et al., 2011). It remains to be seen how this might interact with the presence or absence of meaningful visuospatial context. Experiment 1 therefore examined to what extent the VSB advantage varies with concurrent verbal activity during retention. Scaffolding of verbal memory performance using visuospatial information as indexed by the VSB effect might not be well-suited to longer retention, meaning that participants rely more on verbal coding even in the keypad condition. Preventing this through application of articulatory suppression to the delay period might therefore encourage use of alternative forms of coding where available, thus boosting the VSB effect. Similarly, absence of any useful spatial information in the single location condition likely increases reliance on verbal coding and rehearsal. Therefore, we predicted a larger bootstrapping effect when a simple verbal task was performed during maintenance.

### Method

#### Participants

Thirty participants (21 females and nine males, mean age 20.23 years, range 18–24 years) took part in a single 30-min session.

The key outcome of interest in each of the current experiments was the difference between single location and keypad display conditions. A pilot study^1^ produced an effect size of *d* = .59 over a 5-s delay for this comparison, with G*Power (Faul et al., 2009) indicating a required sample size of *n* = 25 to detect this effect (α = .05, 80% power).

#### Design, materials and procedure

This experiment implemented a repeated-measures 2 x 2 design, manipulating display (single location vs. keypad) and delay task (no task vs. AS). The two delay-task conditions were implemented in separate counterbalanced blocks, while display type trials were randomly intermixed. There were 15 trials in each cell of the design. The primary dependent variable was the proportion of digits correctly recalled per sequence, with scoring implemented on a strict serial position basis.

Materials and procedure were generally closely based on those implemented in Allen et al. (2015). Testing was controlled on a 13-in. MacBook, using a program written in SuperCard (Version 4.7; Solutions Etcetera, Hanover Park, IL, USA). Each session began with a span task to ascertain experimental sequence length to use for each participant. This was based on the single location condition (Fig. 1, see below), using a 1-s retention interval. This span task involved one practice trial at sequence length 2, followed by sequence sets (starting at length 2) that increased in length by one digit every third trial. The task continued until participants failed to correctly recall either of the two sequences at a given length, with span identified as the longest length at which both sequences were correct.

This was followed by the experimental phase, with all sequences titrated to each individual participant’s span length (mean = 6.6, SE = .18, range 4–8). Each trial was started by participants pressing a space bar and began with a fixation cross presented centrally for 500 ms, a 250-ms blank-screen delay, and then the to-be-remembered digit sequence. Digits were presented in black 36-pt Arial font on a green background within a blank square outline measuring 60 x 60 pixels. Each display was presented for 500 ms and separated by a 500-ms blank screen interstimulus interval. For the single location condition, each item was presented in isolation at screen centre. For the keypad condition, all ten digits (0–9) were presented in a familiar keypad layout (Fig. 1), with 12 pixels separating each square.

Completion of the presented sequence was followed by a 5-s blank screen delay. In the no-task condition, participants were not required to do anything during this period. In the AS task condition, they were required to repeatedly articulate the phrase ‘*coca-cola’* at a rate of approximately one repetition per second (as in Allen et al., 2015).

The response phase was then signaled by a 500-ms tone played through the laptop speakers. On hearing this cue, participants attempted to verbally recall the full sequence in its original order, substituting ‘blank’ for serial positions they could not retrieve.

### Results

Outcomes were examined with repeated-measures ANOVA followed by paired-samples t-tests and effect size estimates (Cohen’s *d*) concerning the size of the VSB advantage (i.e., single location vs. keypad display) for each task condition. Bayesian ANOVA and t-tests were also carried out (using default priors within JASP 0.16.3). Bayes Factors (BFs) provide an estimate of the strength of evidence for the data under the null and alternative hypotheses. For ANOVA, these correspond to BF_incl_, i.e., the strength of evidence for the inclusion of each factor and interaction in the model. For t-tests, BF_10_ are reported, indicating evidence for the presence of an effect. BF < 1 indicates support for the null hypothesis, and BF > 1 support for the alternative hypothesis. Bayes Factors represent a continuous estimate of strength of evidence, but to aid interpretation we adopt the widely used classification scheme in which BF 1–3 equates to anecdotal evidence, BF 3–10 as moderate evidence, and BF > 10 as strong evidence (Jeffreys, 1961; Lee & Wagenmakers, 2013).

**Fig. 2:**
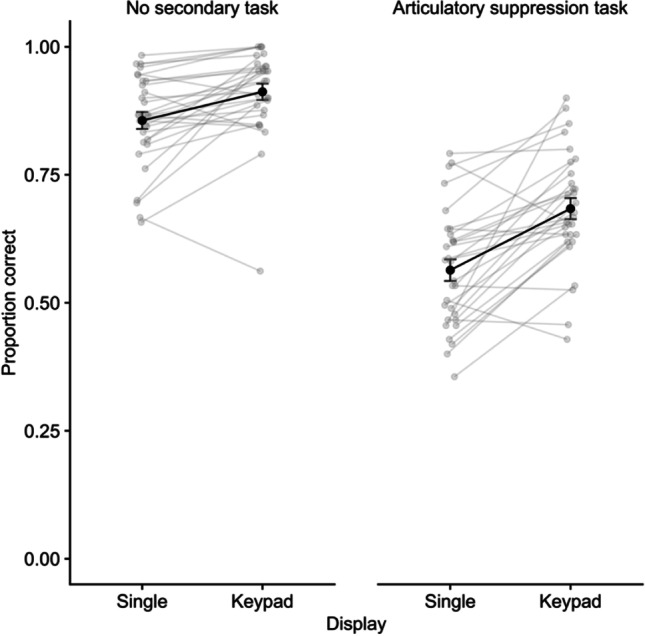
Mean proportion correct for digit recall in Experiment 1. Error bars show standard error, and grey points illustrate individual participants

Performance on the digit recall task is illustrated in Fig. 2. A repeated-measures ANOVA on the proportion of digits correctly recalled per sequence indicated significant effects of display, *F* (1,29) = 50.50, *p* < .001, η^2^p = .64, *BF* > 10,000, delay task (AS), *F* (1,29) = 338.28, *p* < .001, η^2^p = .92, *BF* > 10,000, and the interaction, *F* (1,29) = 5.87, *p* = .022, η^2^p = .17, *BF* = 6.76. Comparing display types for each task condition, an advantage for keypad over single location recall emerged in the no-task condition, *t*(29) = 3.82, *p* < .001, *d* = .70, *BF* = 48.41, and the AS condition, *t*(29) = 5.72, *p* < .001, *d* = 1.04, *BF* = 5557, though this difference was larger in the latter case.

### Discussion

Experiment 1 demonstrated several outcomes. Firstly, the digit recall advantage for keypad over single location presentation remained over delays of 5s, replicating outcomes from the pilot study and extending previous demonstrations that used shorter (typically 1s) intervals. Secondly, a simple AS task performed during the delay period substantially reduced performance in both display conditions, suggesting verbal rehearsal to be a commonly employed strategy in retaining digits following either form of presentation. These findings are in line with those of Allen et al. (2015) and Allan et al. (2018), and with the observation from Yousif et al. (2021) that participants often self-reported using rehearsal even when spatial structure was available to benefit performance. However, the extent to which participants rely on such a maintenance strategy appears to somewhat vary with presentation format, as this AS task served to increase the size of the keypad advantage.

This interactive pattern replicates and extends those of Allen et al. (2015, Experiment 1; see also Allan et al., 2018). Thus, enabling VSB through the provision of a spatial configuration during presentation serves to reduce the disruption caused by concurrent verbal processing during both encoding and subsequent maintenance. In the present context, given the large negative impacts on recall caused by performance of the verbal suppression task for 5 seconds post-encoding, providing a meaningful spatial layout may mitigate against this by enabling verbal recall via a non-verbal path.

## Experiment 2

What processes, if any, are important in supporting the continued maintenance of the VSB effect during retention? Allen et al. (2015) found that spatial tapping applied during encoding removed the keypad advantage (Experiment 2), but this effect survived when the tapping task was moved to the recall phase (Experiment 3). Although this suggests that spatial processing is important in deriving the generation and maintenance of VSB during encoding, but not during recall, it does not separate out processes that might contribute during encoding vs. retention. Experiment 2 was carried out to examine how spatial processing might play a role in maintaining the keypad advantage over time.

When maintaining information over brief delays, there is evidence to suggest that visuospatial processing and attention play an active role. Delay-based distraction tasks that require eye movement disrupt memory for location information (Pearson & Sahraie, 2003; Postle et al., 2006), indicating that oculomotor control processes may support the rehearsal of rehearsal of location-specific representations. Use of the ‘looking-at-nothing’ paradigm has also suggested that participants tend to saccade to relevant but now empty locations when carrying out memory tasks (Ferreira et al., 2008; Morey et al., 2018; Tremblay et al., 2006). This may capture how location-related items are refreshed in working memory (though see Loaiza & Souza, 2022, for recent evidence that participants do not necessarily spontaneously engage in this behaviour during maintenance). Spatial processing resources may also be useful more generally in helping maintain location-relevant information, even if this does not explicitly involve saccades to occupied locations during maintenance.

Following on from Allen et al. (2015), spatial tapping was used as the delay task in Experiment 2. This task is assumed to load on spatial processing. When performed as a secondary task after stimulus encoding, it has been shown to disrupt spatial memory tasks such as Corsi blocks (Della Sala et al., 1999; Pearson & Sahraie, 2003) mental rotation and synthesis (Logie & Salway, 1990; Pearson et al., 1999) and memory for movements (Smyth & Waller, 1998; Smyth & Pendleton, 1989). In the context of VSB, Allan et al. (2018) suggested that spatial tapping does not prevent spatial trace formation, but rather disrupts or prevents its use in supporting performance. We predicted that, if the bootstrapping effect requires continued maintenance of digits within spatial configurations after offset, it should be reduced or even abolished by performance of a spatial tapping task during retention.

### Method

#### Participants

Thirty participants (22 females and 8 males, mean age = 19.83, range 18-26) took part in a single 30-minute session.

#### Design, materials, and procedure

These were generally closely based on Experiment 1, with a repeated measures 2x2 design, manipulating display (single location vs. keypad) and delay task (no task vs. spatial tapping). The two delay-task conditions were implemented in separate counterbalanced blocks, while display type trials were randomly intermixed. There were 15 trials in each cell. Sequences were titrated to each individual participant’s span length (mean = 6.57, SE = .21, range 5-9).

The digit recall task was implemented in the same way as Experiment 1, with a 5s blank screen retention interval between the final item in the sequence and the verbal recall phase. Spatial tapping was implemented as in Allen et al. (2015) but was limited to the 5s retention interval (rather than being performed during encoding or recall phases). Four black felt pads (each 2.8 cm^2^) were attached to a secure base and arranged in a cross formation (see Fig. 1c) and placed out of view behind a screen (though still in easy reach of the participant). Participants were asked to carry out a regular and repeated *up-down–left-right* movement on the pads, at approximately two taps per second. They were first permitted to familiarize themselves with the configuration and pattern of motion before the first of the tapping conditions. The spatial array was then placed out of view behind the screen during task performance to minimize visual disruption and emphasize the spatial nature of the task.

Participants were asked to perform the spatial tapping task from the end of sequence presentation through to the recall phase. As in Experiment 1, the end of the delay phase and commencement of digit recall was signalled with an auditory tone.

### Results

**Fig. 3:**
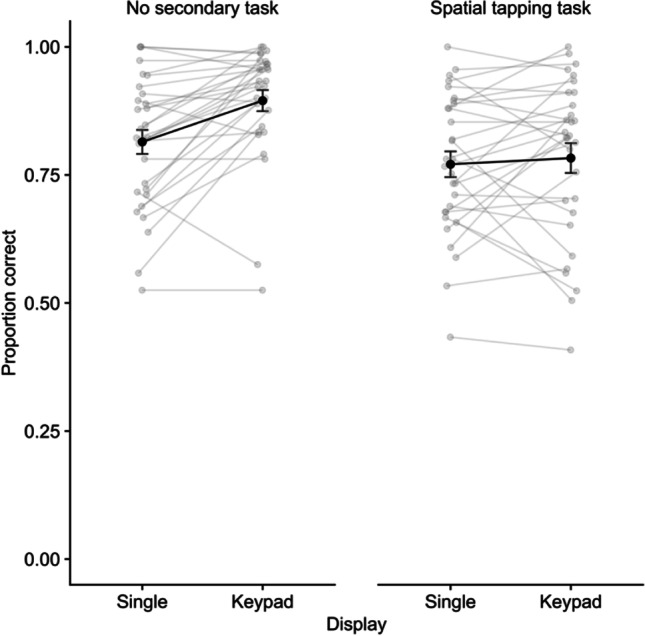
Mean proportion correct for digit recall in Experiment 2. Error bars show standard error, and grey points illustrate individual participants

Performance on the digit recall task is illustrated in Fig. 3. A repeated measures ANOVA indicated significant effects of display, *F* (1,29) = 9.89, *p* = .004, η^2^p = .25, *BF* = 6.71, delay task (ST), *F* (1,29) = 40.95, *p* < .001, η^2^p = .59, BF = 2625, and the interaction, *F* (1,29) = 5.77, *p* = .023, η^2^p = .17, *BF* = 7.46. Comparing display types for each task condition, an advantage for keypad over single location recall emerged in the no-task condition, *t*(29) = 4.12, *p* < .001, *d* = .77, *BF* = 121, but not the spatial tapping condition, *t*(29) = .55, *p* = .58, *d* = .10, *BF* = .224.

### Discussion

Spatial tapping during maintenance reduced digit recall performance, indicating a role for spatial processing in retaining visually presented verbal sequences. This disruption was not of the same apparent magnitude as that caused by AS in Experiment 1, illustrating the verbal nature of the primary task, and the effectiveness of these dual-task manipulations in differentiating between different processing components. However, in contrast to Experiment 1, the disruption caused by this maintenance-based spatial task served to remove the recall advantage for keypad over single-location displays. This extends the findings of Allen et al. (2015, Experiment 2); the beneficial effects of presenting verbal material within a familiar spatial configural context during encoding continues over 5-s retention intervals but appears to be vulnerable to dual-task interference from a spatial task during this maintenance phase of the task.

## Experiment 3

This experiment aimed to replicate and extend the findings of Experiment 2 to the use of a different visuospatial task applied during the delay period. We used a spatial fit task adapted from Vergauwe et al. (2009, 2010, 2012; Langerock et al., 2014; Roth & Hellige, 1998; Rybash & Hoyer, 1992). Vergauwe et al. (2009) showed that this task can disrupt memory for visual and spatial material when interspersed with stimulus presentation during encoding. Increasing the cognitive load imposed by this task by requiring more judgements within a limited retention interval also appears to impact on maintenance of visually and auditorily presented verbal material (Vergauwe et al., 2010, 2012). Of relevance to the current study, Langerock et al. (2014) compared the impacts of different dual tasks on memory for verbal or cross-modal (verbal-spatial associations, i.e., letters presented in different locations) material. They found that the spatial fit task, when interspersed with to-be-remembered stimuli during presentation, had a larger impact on cross-modal associations than on verbal-only memory.

Building on the outcomes of Experiment 2, we therefore predicted that the use of a different spatial task during maintenance would lead to the same broad pattern of outcomes, namely performance decrement in both display conditions, and the reduction or removal of the VSB advantage. We also measured performance in the spatial fit task. If retaining a multi-modal representation that includes spatial configural information draws on spatial processing resources, performance in the spatial fit task should be less effective when retaining keypad rather than single-location displays.

### Method

#### Participants

Thirty-one participants initially took part in this experiment. However, three participants failed to respond on > 50% of subtrials in the secondary visuo-spatial task performed during the delay period and so were removed from all analyses. Thus, the final sample included 28 participants (23 females, five males, mean age = 18.94 years, range 18–21 years) who took part in this experiment, within a single 30-min session.

#### Design, materials and procedure

This was closely based on the previous experiments, implementing a repeated-measures 2 x 2 design, with display (single location vs. keypad) and delay task (no task vs. visuospatial task) as factors. The two concurrent task conditions were implemented in separate counterbalanced blocks, while display type trials were randomly intermixed. There were 15 trials in each cell. Sequences were titrated to each individual participant’s span length (mean = 6.39, SE = .19, range 5–9).

For the spatial fit task, participants completed three subtrials (each lasting 1,500 ms) within the 5-s delay phase of each trial (equating to one response required every 1.67 s, i.e., a low-medium load, based on Langerock et al., 2014). On each subtrial, they were presented with a box on-screen containing a central horizontal line with two black dots located either above or below it (see Fig. 1d). The length of the line varied between subtrials so that it could sometimes fit in the gap between the dots, with 50% probability. There were 24 variants of the display in total, sampled pseudo randomly with replacement across the experiment. Participants were required to press the ‘>’ key on the keyboard if they thought the line fitted, and the ‘/’ key if not. They were required to press the key within the 1,500-ms time window on each subtrial.

As in the previous experiments, participants were asked to perform this task from the end of sequence presentation through to the recall phase, with the end of the delay phase and commencement of digit recall signalled with an auditory tone.

#### Data processing

Accuracy and reaction time for the visuospatial delay task were recorded and analysed in this experiment. As already noted, three participants in the initial sample failed to respond on > 50% of subtrials in this task, and so were removed from all analyses. In addition, we also removed all three subtrials from a given trial when the participant had either produced less or more than three responses during that specific delay period, resulting in removal of 12% of the visuospatial task data. The resulting analysis is focused on accuracy (proportion correct) and reaction time for correct responses.

### Results

**Fig. 4:**
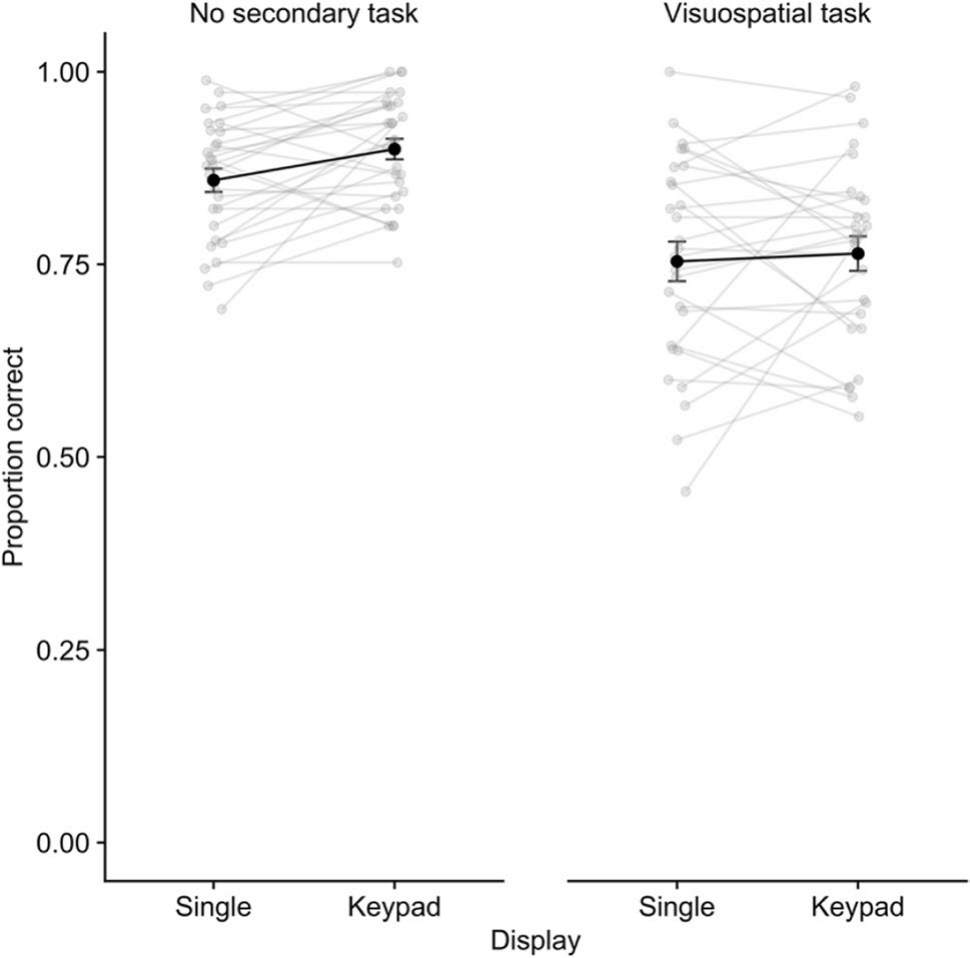
Mean proportion correct for digit recall in Experiment 3. Error bars show standard error, and grey points illustrate individual participants

Performance on the digit recall task is illustrated in Fig. 4. A repeated-measures ANOVA on the proportion of sequences indicated no significant effect of display, *F* (1,27) = 2.93, *p* = .099, η^2^p = .10, *BF* = .75, but a significant effect of delay task (VS), *F* (1,29) = 40.95, *p* < .001, η^2^p = .59, *BF* > 10,000. The interaction was not significant, *F* (1,27) = 1.58, *p* = .22, η^2^p = .06, *BF* = .715. Given our a priori hypotheses, and the outcomes from the first two experiments, we continued by comparing display types for each task condition. An advantage for keypad over single location recall emerged in the no-task condition, *t*(27) = 2.83, *p* = .009, *d* = .53, BF = 5.15, but not the visuospatial task condition, *t*(27) = .45, *p* = .66, *d* = .08, *BF* = .220.

**Fig. 5:**
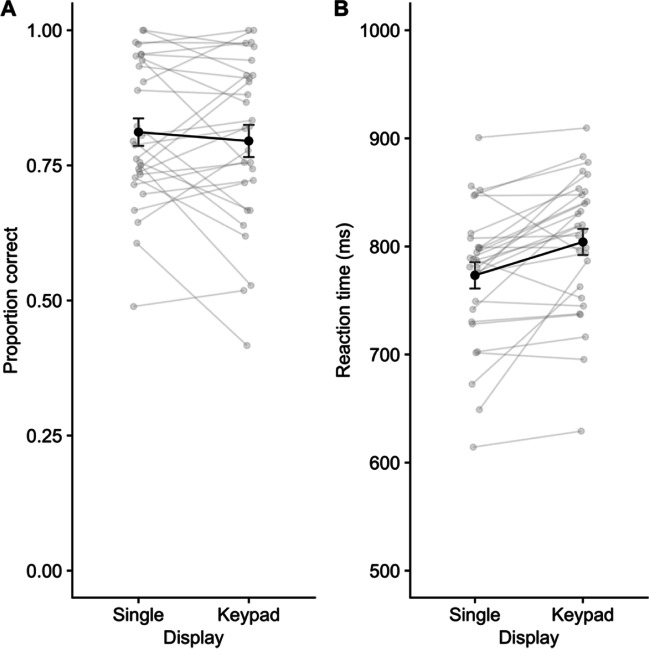
**A** Mean proportion correct. **B** Mean reaction time on the visuospatial delay task in each of the display conditions in Experiment 3. Error bars show standard error, and grey points illustrate individual participants

Performance on the VS line judgment task is illustrated in Fig. 5. There was no difference between digit display types for VS task accuracy, *t*(27) = .83, *p* = .414, *d* = .16, *BF =* .28. However, for reaction time, participants were faster to respond during single location digit recall, relative to keypad digit recall, *t*(27) = 3.91, *p* < .001, *d* = .74, *BF =* 56.39.

### Discussion

Performing a visuospatial task during the delay period reduced performance in the digit recall task. Regarding the effects of this task on maintenance of digits using the different configurations, some caution should be exercised given there was no significant main effect of display type or interaction with delay task. That said, there was evidence in this experiment for relatively greater mutual interference in the keypad condition. Firstly, the VSB advantage was present in the no-task condition (replicating Experiments 1 and 2), and this was removed in the visuospatial task condition. This pattern of effects mirrors those observed in Experiment 2 using spatial tapping. Secondly, inspection of performance in the delay task indicated that mean reaction time was significantly increased on trials where digits had been presented within a keypad configuration, relative to a single location. Thus, participants were slower to make line judgements when retaining digits in spatial configurations, and making these judgements removed the recall advantage provided by such configurations. Thus, maintaining visually presented digit sequences for verbal recall does appear to draw on visuospatial processing, and this is particularly the case when the digits are encountered in a familiar spatial configuration.

## Cross-experiment analysis

Data from the first three experiments were combined to further establish the extent to which patterns of display effects change across concurrent task conditions. The key focus here is whether task and display interact when combining experiments, and whether this interacts with experiment (which denotes comparison of different concurrent task manipulations).

An initial 2 x 2 x 3 mixed ANOVA across Experiments 1–3 with experiment as a between-subjects factor indicated significant effects of display, *F* (1,85) = 43.40, *p* < .001, η^2^p = .34, *BF* > 10,000, delay task, *F* (2,85) = 43.40, *p* < .001, η^2^p = .80, *BF* > 10,000, but not the interaction, *F* (2,85) = .57, *p* = .45, η^2^p = .01, *BF* = .15. There was a main effect of experiment, *F* (2,85) = 4.39, *p* = .015, η^2^p = .09, *BF* = 3.48. Experiment also interacted with display, *F* (2,85) = 5.20, *p* = .007, η^2^p = .11, *BF* = 4.18, task, *F* (2,85) = 45.52, *p* < .001, η^2^p = .52, *BF* > 10,000, and more importantly, there was a three-way interaction between display, task, and experiment, *F* (2,85) = 6.76, *p* = .002, η^2^p = .14, *BF* = 57.40.

Given this three-way interaction, further sets of 2 x 2 x 2 analyses were carried out for each experiment. Comparing AS and ST (Experiment 1 vs. Experiment 2) indicated significant effects of display, *F* (1,58) = 48.74, *p* < .001, η^2^p = .46, *BF* > 10,000, delay task, *F* (1,58) = 328.08, *p* < .001, η^2^p = .85, *BF* > 10,000, but not the interaction, *F* (1,58) = .01, *p* = .91, η^2^p = .01, *BF* = .20. There was a main effect of experiment, *F* (1,58) = 5.59, *p* = .021, η^2^p = .09, *BF* = 2.82. Experiment interacted with display, *F* (1,58) = 4.70, *p* = .034, η^2^p = .08, *BF* = 1.17, and task, *F* (1,58) = 95.28, *p* < .001, η^2^p = .85, *BF* > 10,000. Crucially, there was a three-way interaction between display, task and experiment, *F* (1,58) = 11.61, *p* = .001, η^2^p = .17, *BF* = 123.62.

Comparing AS and VS tasks (Experiment 1 vs. Experiment 3) indicated significant effects of display, *F* (1,56) = 34.86, *p* < .001, η^2^p = .38, *BF* > 10,000, delay task, *F* (1,56) = 310.77, *p* < .001, η^2^p = .85, *BF* > 10,000, but not the interaction, *F* (1,56) = .89, *p* = .35, η^2^p = .02, *BF* = .32. There was a main effect of experiment, *F* (1,56) = 9.47, *p* = .003, η^2^p = .15, *BF* = 11.79. Experiment interacted with display, *F* (1,56) = 10.69, *p* = .002, η^2^p = .16, *BF* = 9.78, and task, *F* (1,56) = 41.89, *p* < .001, η^2^p = .43, *BF* > 10,000, and there was again a three-way interaction between display, task and experiment, *F* (1,56) = 6.90, *p* = .011, η^2^p = .11, *BF* = 7.45.

Finally, comparing ST and VS tasks (Experiments 2 and 3) indicated significant effects of display, *F* (1,56) = 11.75, *p* = .001, η^2^p = .17, *BF* = 19.48, delay task, *F* (1,56) = 95.84, *p* < .001, η^2^p = .63, *BF* > 10,000 (with the VS task in Experiment 3 having a larger impact than the ST task in Experiment 2), and the display x task interaction, *F* (1,56) = 6.91, *p* = .011, η^2^p = .11, *BF* = 9.19. Following this up, there was a keypad advantage under no-task conditions, *t*(57)=4.99, *p* < .001, *d* = .65, *BF* = 2885, but not with the ST/VS conditions, *t*(57)=.48, *p* = .477, *d* = .09, *BF* = .183. There was no main effect of experiment, *F* (1,56) = .02, *p* = .901, η^2^p = .00, *BF* = .467, but an interaction with task, albeit weakly supported by Bayesian analysis, *F* (1,56) = 4.40, *p* = .041, η^2^p = .07, *BF* = 1.36. There was no interaction between experiment and display, *F* (1,56) = 1.01, *p* = .319, η^2^p = .02, *BF* = .272, and no three-way interaction, *F* (1,56) = 1.04, *p* = .312, η^2^p = .02, *BF* = .334.

These cross-experiment analyses confirm the patterns observed in the separate experiments, with the delay-based task required in each experiment changing the size and presence of the keypad display advantage. Verbal activity during a maintenance period (articulatory suppression in Experiment 1) increases the bootstrapping effect, whereas a concurrent spatial or visuospatial activity (spatial tapping in Experiment 2 and line judgement in Experiment 3) abolishes it.

## Experiment 4

This final experiment examined evidence for a role of modality-general attentional resources during maintenance of digit sequences following presentation using each of the two display types. Such resources are important in working memory encoding and maintenance, as captured by theoretical frameworks that incorporate some form of executive control (e.g., Baddeley et al., 2021; Barrouillet & Camos, 2021; Cowan et al., 2021; Vandierendonck, 2021). This has been shown using dual-task methodology in the context of verbal serial recall (Atkinson et al., 2021; Baddeley et al., 2009), and of relevance to the current work, visuospatial bootstrapping (Calia et al., 2019). Calia et al. found that recall of visually presented digit sequences was substantially reduced when performing a more complex verbal task during encoding. However, the observed advantage for keypad over single-location displays did not reduce, and in fact increased in size, when participants were attempting to perform the more complex task. This would suggest that, rather than incorporating beneficial visuospatial information into working memory relying on general attentional control, this is an implicit and automatic process; when such information is not available, successful performance is perhaps more dependent on executive support.

Calia et al.’s findings to some extent fit with those of Baddeley et al. (2009) in indicating facilitative effects derived at least in part from stored knowledge in LTM that do not hinge on executive resource availability. What might we expect if the load was shifted to a slightly extended maintenance period? On the one hand, we might see a similar finding to that observed with encoding-based tasks, with VSB surviving unabated and possibly showing some evidence of protection from executive load costs. Alternatively, retaining information over time in an integrated or associated form may draw on general attentional control. Thus, we may see reduction or removal of the VSB effect when attentional load is manipulated during maintenance. There is no existing evidence that directly speaks to this, though Zokaei et al. (2014) found that the probability of binding errors in memory for conjunction stimuli increased with the presence and difficulty of an intervening visual search task.

We used simple and complex verbal tasks as implemented in the context of VSB by Calia et al. (2019), involving either stating a list of days or months (simple task), or an alternating list of days and months (complex task). It is assumed that both tasks load on verbal processing, but that the more complex task places an increased load on general attentional resources. We predicted that serial recall performance would be reduced overall in the complex-task condition, extending findings of Calia et al. from encoding to the maintenance period, and in line with a range of research indicating working memory storage draws on executive control (e.g., Allen et al., 2017; Morey & Cowan, 2005). Finally, if the apparent automaticity of the VSB advantage observed by Calia et al. (2019) during encoding extends across a longer maintenance period, the magnitude of this effect should survive or even increase with a more complex concurrent task. Alternatively, a reliable reduction in the size of the VSB effect with a more complex concurrent task would indicate that it draws on general executive resources.

### Method

#### Participants

Thirty participants (27 females, three males, mean age = 19.37 years, range 18–22 years) took part in a single 30-min session.

#### Design, materials and procedure

This experiment again implemented a repeated-measures 2 x 2 design, manipulating display (single location vs. keypad) and delay task (simple verbal task vs. complex verbal task). The two concurrent task conditions were implemented in separate counterbalanced blocks, while display type trials were randomly intermixed. There were 15 trials in each cell. Sequences were titrated to each participant’s span length (mean = 6.5, SE = .20, range 5–9).

For each delay task, the start point was presented on-screen after digit sequence offset. In the simple task, participants were provided with a day or month (e.g., *Tuesday* or *September*) and asked to state out loud the following days or months within an allotted time (e.g., “*Wednesday, Thursday, Friday, Saturday…”)*. In the complex task, a day and month were provided (e.g., *Tuesday – September*), and participants listed the subsequent iterations in alternating order (e.g., “*Wednesday, October, Thursday, November…”*).

### Results

**Fig. 6:**
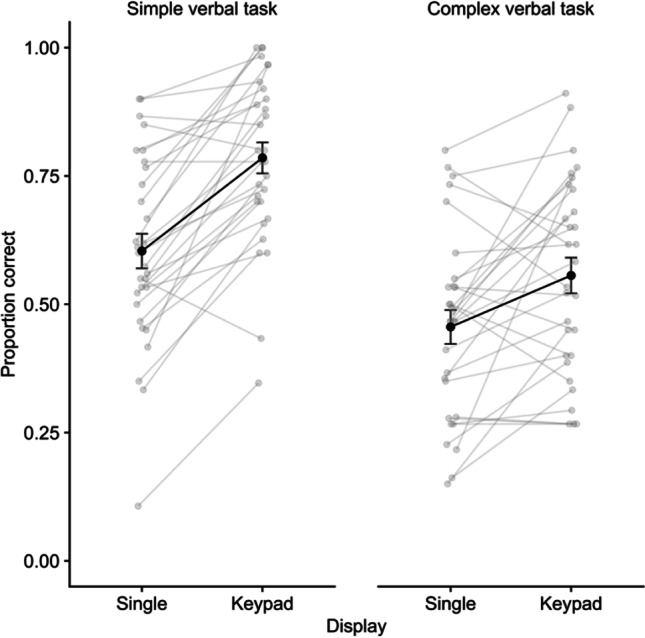
Mean proportion correct for digit recall in Experiment 4. Error bars show standard error, and grey points illustrate individual participants

Mean performance in the digit recall task is illustrated in Fig. 6. A repeated-measures ANOVA on proportion of sequences correctly recalled indicated significant effects of display, *F* (1,29) = 31.05, *p* < .001, η^2^p = .21, BF = 4199, with keypad recall (M = .67, SE = .03) superior to single location recall (M = 53, SE = .03). There was also an effect of delay task (CE), *F* (1,29) = 78.53, *p* < .001, η^2^p = .73, BF > 10,000, with digit recall accuracy superior in the simple task (M = .70, SE = .03) relative to the complex-task conditions (M = .51, SE = .03). The interaction was also significant, *F* (1,29) = 8.58, *p* = .007, η^2^p = .23, *BF* = 7.35. Comparing display types for each task condition, an advantage for keypad over single-location recall emerged in the simple task condition, *t*(29) = 7.03, *p* < .001, *d* = 1.28, BF > 10,000, and in the complex task, *t*(29) = 3.18, *p* = .004, *d* = .58, *BF* = 11.06, though it was larger in the former case.

**Fig. 7:**
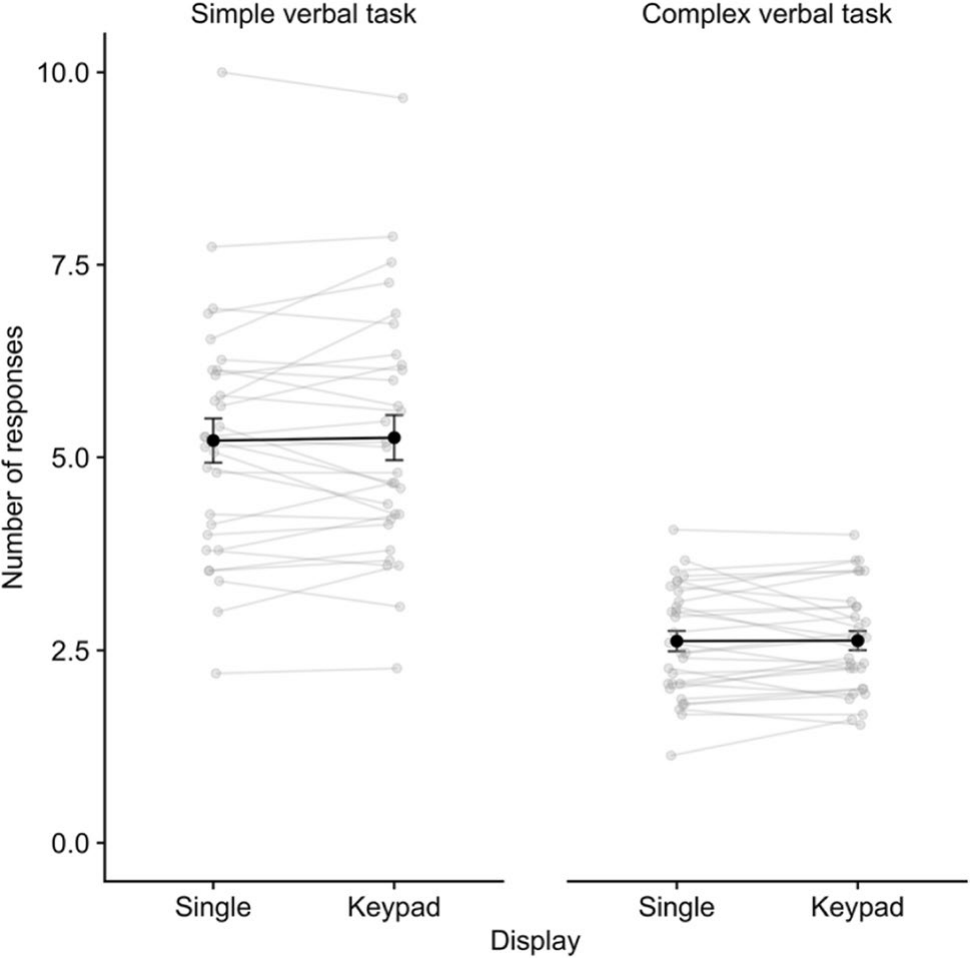
Mean number of responses in the simple and complex verbal delay tasks for each display type in Experiment 4. Error bars show standard error, and grey points illustrate individual participants

For the verbal delay task, the number of responses made on each trial was examined (see Fig. 7). A 2 x 2 ANOVA indicated a significant main effect of task,* F* (1,29) = 97.14, *p* < .001, η^2^p = .77, BF > 10,000, with more responses made for the simple (M = 5.24, SE = .22) relative to the complex task (M = 2.62, SE = .22). There was no effect of digit display or the interaction (*F* < .15, *p* > .70, η^2^p < .01, *BF* < .3).

### Discussion

Recall was again improved following keypad presentation, and this effect was present in both delay-task conditions. However, there was some evidence that the size of this effect was reduced with a more complex and attentionally demanding verbal task was applied during retention. Calia et al. (2019) found that the same concurrent tasks applied during encoding did not reduce the visuospatial bootstrapping effect, and in fact appeared to somewhat increase the advantage for typical keypad over single-location displays in one of the outcome measures (the same one used here). This would indicate that generating the keypad advantage during encoding is relatively automatic, in line with the notion of initial binding processes that do not load on attentional control (Allen et al., 2006; Baddeley et al., 2009, 2011). However, the present study suggests an executive component in maintaining the resulting representations. This does not appear to be critical to the survival of the VSB effect though, as it was observable in the complex-task condition, albeit somewhat reduced in size. Thus, continued availability of executive resources is helpful but not crucial for maintaining the VSB advantage.

## General discussion

The current series of experiments aimed to explore how information across verbal and visuospatial domains might be maintained over time in working memory to benefit verbal recall performance. This was explored in the context of the visuospatial bootstrapping (VSB) effect and using distractor tasks that were applied during a short retention interval to target different dimensions of working memory processing. Extending previous demonstrations of the VSB effect, all four experiments showed a recall advantage for keypad over single location presentation in the no-task condition, demonstrating that this effect can survive maintenance intervals of up to 5 s.

However, this effect shifted depending on what the participant was asked to do during these intervals. Application of a simple verbal (AS) task during maintenance in Experiment 1 substantially affected performance for both display types, but also served to increase the size of the VSB effect. These findings represent an extension of that observed during encoding (Allen et al., 2015, Experiment 1; Allan et al., 2018) to the maintenance interval. In contrast, when a spatially oriented tapping task was instead required during maintenance, the reverse pattern to AS was observed, with the verbal recall advantage for keypad displays being abolished (Experiment 2), again extending findings reported by Allen et al. (2015, Experiment 2) from encoding to retention. Experiment 3 extended exploration to a visuospatial interference task, with findings broadly in line with those using a purely spatial task in Experiment 2. Although the overall display by task interaction was not supported, the VSB effect was removed when participants performed a visuospatial task during maintenance. Furthermore, mean reaction time in this secondary task was slower on trials where the original verbal sequence had been presented in a keypad configuration, compared to the single location condition; provision of a visuospatial configuration in an otherwise purely verbal task slows later visuospatial processing. Finally, Experiment 4 required the performance of a verbal task during retention in all conditions, manipulating the general attentional demands of this task. Performance substantially declined for both display types, but this decline was relatively larger for the keypad display condition, with the VSB advantage being reduced (though not removed) as a result. Calia et al. (2019) used the same interference tasks during encoding rather than maintenance, finding that resulting disruption caused by the more demanding task was reduced rather than increased in the keypad condition, using the same outcome measure (proportion correct per sequence) that was implemented here. Overall, the present study demonstrates that (1) provision of visuospatial information during encoding can continue to benefit verbal working memory over short retention intervals; (2) these continuing benefits interact with domain-specific (i.e., verbal and visuospatial) processing during maintenance in the same way as previously observed in the context of encoding (Allen et al., 2015); and (3) this does not apply for domain-general attentional control resources, which appear to differ between encoding and maintenance (Calia et al., 2019). Thus, these patterns of findings show how encoding and maintenance in working memory both overlap and differ in the constituent processing components that are involved, at least in the present task context.

Disruption from domain-specific secondary tasks likely reflects both interference with the representation itself, and with rehearsal mechanisms available to support continued maintenance and prevent loss from working memory. Verbal rehearsal has long been explored as a potentially effective strategy for maintenance over at least the short term and the imposition of articulatory suppression is assumed to prevent or at least disrupt this process (e.g., Baddeley et al., 1975; Murray, 1968; Vergauwe et al., 2014; but see Oberauer, 2019, for a different view of rehearsal as a maintenance strategy). Maintenance of verbal material over time does indeed appear to draw on domain-specific verbal processing (e.g., Jarrold et al., 2011). The findings from Experiment 1 show that verbal representation and rehearsal are indeed important components of maintenance for serial recall of verbal material that was originally visually presented. However, the observation of a larger display effect following AS interference during retention might reflect a reduced reliance on verbal processing in the keypad condition when visuospatial information is also available. It may also indicate how the initial provision of this visuospatial information at encoding serves to provide an alternate route to successful retrieval when verbal information is no longer readily available.

Spatial processing resources are important in ensuring participants can maintain such information over time (e.g., Hale et al., 1996; Logie & Marchetti, 1991). Experiments 2 and 3 show that this impacts on the ability to benefit from spatialization in an otherwise verbal recall task. Maintaining the beneficial effect of visuospatial information on subsequent verbal recall appears to draw on visuospatial processing resources, with effects evident on both the primary working memory task (in Experiments 2 and 3) and the secondary interference task in Experiment 3. It is also worth noting that Allen et al. (2015, Experiment 3) found no interactive impact of ST when it was instead applied at the recall phase. Taken in conjunction, encoding and maintenance appear to be distinct from explicit recall in the demands placed on spatial processing.

Interference effects on maintenance may at least partly reflect domain-specific rehearsal mechanisms that would otherwise support continued storage of those elements. Participants may engage in mental refreshing and rehearsal of the occupied locations and the path through these locations during the maintenance interval. There is some evidence from the ‘looking-at-nothing’ phenomenon that participants’ eye movements fixate to locations associated with previously presented information (Richardson & Spivey, 2000). Applying this to a working memory context, Pearson and Sahraie (2003) suggested a role for oculomotor control processes during rehearsal of location-specific representations in working memory during retention, and Postle et al. (2006) reported evidence showing that working memory for spatial locations could be disrupted by requiring eye movement during the delay period of their recognition task. Tremblay et al. (2006) also found that eye-movement fixation patterns indicated rehearsal of visuospatial information during maintenance. However, Loaiza and Souza (2022) reported that spontaneous eye fixations only revisited previously occupied locations during retention when location marker ‘placeholders’ were present, and spontaneous fixations did not predict recall precision (though they were related to item cueing). The presence of reliable and observable shifts in spatial attention during maintenance, and a meaningful pattern of relationships with memory performance, likely depends in part on the nature of the primary task, which in each case differs from that employed in the present study. Candidate explanations for the evidence of visuospatial disruption during maintenance that is presently observed might include fixations to previously occupied locations, as well as direct interference with the memory representation, and disruption of refreshing. Future work will need to pick apart these possible accounts.

The final experiment in the series indicated a substantial reduction in task accuracy when a more demanding verbal task was performed during retention, showing again the important role for modality-general executive control resources in working memory maintenance (e.g., Allen et al., 2017; Barrouillet & Camos, 2021; Morey & Cowan, 2005). Furthermore, the significant reduction but not removal of the VSB effect under such conditions would suggest that attentional resources contribute to, but are not critical for, the survival of this benefit over time. Calia et al. (2019) concluded that encoding of verbal and visuospatial information to support VSB is relatively automatic, in line with other evidence that initial binding in working memory does not have an attentional cost (e.g., Allen et al., 2006, 2012; Baddeley et al., 2011). The current findings would indicate a different story for subsequent maintenance of this information, with attention playing a role in keeping these representations intact and accessible. For example, extended maintenance of associated or bound verbal and visuospatial information might particularly depend on availability of resources to support attentional refreshing, a domain-general process by which representations are reactivated during retention to keep them active and accessible, and prevent loss (e.g., Camos et al., 2018).

An assumption running through much of the work on VSB to date has been that the effect draws on the generation and retention of an integrated representation requiring binding of verbal and visuospatial information and storage in a modality-general form. There is evidence indicating binding in working memory between verbal and spatial information (Elsley & Parmentier, 2009; Morey, 2009; Prabhakaran et al., 2000), and that other dimensions, either within and between modalities, can also be combined to influence working memory task performance (e.g., Allen et al., 2009; Johnson & Allen, 2022; Jones et al., 2013; Quak et al., 2015; Maybery et al., 2009; Thompson & Paivio, 1994; Wang et al., 2015). However, the nature of the representations driving performance and the improvements in VSB remains to be established, particularly regarding how verbal and visuospatial information might directly interact. As noted by Allan et al. (2018), VSB does not require the assumption of a fully integrated set of representations. The basic effect, and the changes with maintenance-based activity observed in the present study, could also be explained via the separate modality-specific storage of verbal and visuospatial information. This would align with dual-coding theory (Paivio, 1991), which states that information from different domains and modalities can interact, but the underlying representations are independent and can be recalled as such if required by the task. From this perspective, verbal and visuospatial codes underlying the VSB effect would operate independently, and each be drawn on to inform recall. The verbal spatial, and visual tasks implemented in Experiments 1–3 of the current study would then be interpreted as disrupting modality-specific storage and associated rehearsal mechanisms. The executive control effect (Experiment 4) might reflect a cost in retaining multiple forms of representational code, or alternatively a greater general cost for holding visuospatial information. In line with this, Morey and Miron (2016) claimed that maintenance of spatial serial order, like the maintenance of visual materials more broadly, draws on general rather than specialized resources, while maintenance of verbal sequences may rely on domain-specific resources. Vergauwe et al. (2014) noted a similar verbal-spatial asymmetry regarding modality-specific and attentional involvement during maintenance.

We would argue, however, that full independence of modality-specific information is unlikely, at least in this task context. As the primary memory task in the current work has an important ordering requirement at recall, it is possible that separate representational streams are connected at least indirectly via serial order coding that is itself modality specific or modality general (e.g., Hurlstone et al., 2014; Logie et al., 2016). It could also be argued that, given that the verbal and visuospatial information that underlies VSB is provided to the participant in integrated form, it would seem at least unlikely for storage to be wholly independent, without any integrative connection between the what and the where. One possibility is that both simple modal representations and more complex multidimensional bound representations are available, respectively within modality-specific and general storage capacities as described for example by Baddeley et al. (2021), and that these might be differentially active depending on the task (Quak et al., 2015), though this comes with a cost to model parsimony. Modality-specific storage and processing mechanisms may also continue to be useful in supporting or ‘backing up’ integrated representations.

Most leading theoretical approaches to working memory allow for the additive contribution of multiple codes from different domains and modalities. Findings in the VSB literature to date, as well as those reported in the current study, do not necessarily support or falsify any one theoretical approach to working memory (Allan et al., 2018), and at least broadly align with any model that allows for the combinatorial effects of multi-domain and multi-modal information (e.g., Baddeley et al., 2021; Barrouillet & Camos, 2021; Cowan et al. 2021; Logie et al., 2021). This might solely involve modality specific storage in the case of a dual-coding interpretation. Within the multicomponent framework, the binding of such verbal and visuospatial information into an integrated representation would encapsulate the sort of function captured by the episodic buffer, a modality-general capacity capable of holding such representations in a consciously accessible form (Baddeley, 2000; Baddeley et al., 2011, 2021). Although there are disagreements regarding the nature of the relationship between working memory and long-term memory, a conceptually similar general storage capacity capable of holding different forms of information is also a key part of alternate theoretical frameworks such as embedded processes (e.g., Cowan, 1999; Cowan et al., 2021; Oberauer, 2002, 2009), in which new associations can be formed between elements from disparate sources that fall within the focus of attention. Indeed, this concept of a focus of attention has recently been incorporated into the multicomponent approach (Baddeley et al., 2021; Hitch et al., 2020; Hu et al., 2014).

Visuospatial bootstrapping serves as an example of a task context in which information from different sources is pulled together to enrich the working memory representation and enhance performance. In that sense, it is analogous to findings from the literature on working memory for instructions, showing that participant enactment and experimenter demonstration serve to boost immediate recall (e.g., Allen & Waterman, 2015; Allen et al., 2020; Jaroslawska et al., 2016; Yang et al., 2014, 2015; for a review, see Allen et al., 2023), likely reflecting recruitment of visuospatial and motoric codes. On the latter point, evidence from dual-task studies on enactment planning has suggested a role for motor coding (Jaroslawska et al., 2018; Li et al., 2022). Relatedly, it is worth noting that the clearest disruption of the display effect in the present study was that produced by spatial tapping in Experiment 2 (see also Allen et al., 2015). Spatial tapping is intended to load on spatial processing, and we assume the broadly similar outcomes to those found using a visuospatial task in Experiment 3 are testament to this. However, there is also a movement component to this task, which may be an additional contributory factor in the overall performance decline and the impact on the keypad display condition. There was no explicit requirement for finger/hand movement from the primary task in the present study, so any possible motor contribution would either be implicitly and automatically cued or arise as part of a strategic response by participants. Examining the various strategic and non-strategic components to such tasks would be a useful exercise in understanding the representational complexity of working memory and would also connect to an emerging literature on strategy in working memory (e.g., Gonthier, 2021; Morrison et al., 2016).

An assumed aspect of the VSB effect is the familiarity of the configuration, and the prior association between digit and location (Darling et al., 2017); providing the same kind of visuospatial framing but in unfamiliar configurations does not lead to the same verbal recall benefits (Darling et al., 2014). This represents one example of how providing contextual information linked to existing LTM representations can enhance working memory, even when the items themselves do not change. Another is the sentence superiority effect, in which working memory for verbal material is improved by being embedded within meaningful syntactic structure (e.g., Allen et al., 2018; Baddeley et al., 2009; Race et al., 2015). In both cases, recall benefits are apparent even though the task does not explicitly require the contextual information and can plausibly be performed without it. This differs from some recent findings in visual working memory (Sobrinho & Souza, 2023), where colour-item congruency only affected performance when it was made explicit to the task. Whether this reflects qualitatively different ways in which working memory and LTM might interact, or varying sensitivity of working memory to the effects of prior association across task types or domains, is a question for future exploration.

The current study adds to a growing evidence base showing that verbal working memory can be enhanced through the provision of helpful, familiar visuospatial context. It aligns with broader literatures showing that working memory can be enhanced through multimodality and scaffolding on prior knowledge. Performance on working memory tasks likely draws on contributions from various sources of information and associated cognitive functions, depending on the materials, the task, and the individual’s ‘repertoire’ (Macken et al., 2015). The present findings demonstrate that provision of visuospatial configurations at encoding can continue to enhance verbal working memory over longer retention intervals. Furthermore, the availability of such information at encoding determines the extent to which modality-specific and general processing resources continue to be required during maintenance.

## Footnotes

1 This pilot experiment (*N* = 30) comparing 1-s and 5-s delays replicated the previously observed advantage for keypad (M = .95, SE = .02) over single location (M = .91, SE = .01) displays (*F* (1,29) = 33.32, *p* < .001, η^2^p = .54, *BF* > 10,000), and did not interact with delay duration (*F* (1,29) = .81, *p* = .375, η^2^p = .03, *BF* = .344).
